# Spontaneous Gastro-Enterostomy Diagnosed by X-Rays

**Published:** 1913-05

**Authors:** 


Spontaneous Gastro-Enterostomy Diagnosed by X-Rays. Douarre of Toulon, *Arch. d'Elect. Med.*, March 10, 1913, mentions the case of a physician aged 73 whose troubles began four years ago, with slight digestive disturbances and occasional cramps which he ascribed to a taenia with which he was infested. The pains became more severe and were accompanied or followed by vomiting. Constipation was present. In May, 1912, he had a terrible nocturnal attack of pain lasting four or five hours. Since this the symptoms were relieved. Physical examination revealed nothing but gastric borborygmi on succussion, slight sensitiveness at the umbilic level, soft abdominal wall without induration or tumor, scybalae in the ascending and descending colon. Immediately after the ingestion of bismuth emulsion the gastric shadow was normal, but, in ten minutes, the mass had passed below the stomach, a streak apparently indicating that it had gone through an opening. The question between gastro-nesteostomy and gastro colostomy, was decided by subsequent views, 6-48 hours after ingestion, showing the gradual, delayed outlining of the colon typically. The lack of marasmus and the persistence of constipation with rather hard faeces also corroborated the opinion that the fistula was between the stomach and the jejunum. Some bismuth remained in the stomach for 24 hours. Reference is made to a similar case of Menuet in the November, 1911, issue of the same journal. (Note: While the observation is interesting and the explanation plausible, there was no section and we rather favor scepticism in extremely rare cases. For example, in a personal case, both ausculatory percussion and X-rays indicated an Hour-glass stomach. Necropsy showed that there was pyloric cancer (the malignant nature of the case had already been diagnosed) and a somewhat dilated duodenum. In any unique case, it is well to assume the attitude of the man who stood before the cage of a rhinoceras for half an hour and then turned away with the remark: "Gosh, there ain't no such critter.")

